# IGF-1 and insulin receptors in LepRb neurons jointly regulate body growth, bone mass, reproduction, and metabolism

**DOI:** 10.1016/j.molmet.2026.102355

**Published:** 2026-03-16

**Authors:** Mengjie Wang, Piotr J. Czernik, Beata Lecka-Czernik, Jennifer W. Hill

**Affiliations:** 1 Center for Diabetes and Endocrine Research, Department of Physiology and Pharmacology, University of Toledo College of Medicine, Toledo, OH, USA; 2 Center for Molecular Psychiatry, Department of Psychiatry and Behavioral Science, University of South Florida, Tampa, FL, USA; 3 Department of Orthopedic Surgery, University of Toledo College of Medicine, Toledo, OH, USA; 4 Department of Obstetrics and Gynecology, University of Toledo College of Medicine, Toledo, OH, USA; 5 Department of Physiology and Pharmacology, University of Toledo College of Medicine, Toledo, OH, USA

**Keywords:** LepRb neurons, IGF1 receptor, Insulin receptor, Reproductive function, Energy balance, Bone metabolism, Hypothalamus, Neuroendocrine regulation

## Abstract

Leptin receptor (LepRb)-expressing neurons integrate metabolic and reproductive signals, yet the role of insulin-like growth factor 1 receptor (IGF1R) signaling within these neurons remains unclear. Because IGF-1 and insulin can partially activate each other’s receptors, we generated mice lacking IGF1R selectively in LepRb neurons (IGF1R^LepRb^) as well as mice lacking both IGF1R and insulin receptor (IR) in LepRb neurons (IGF1R/IR^LepRb^). These models were used to assess body growth, skeletal development, reproductive function, energy balance, and metabolic homeostasis. Deletion of IGF1R alone in LepRb neurons delayed pubertal onset, impaired adult fertility, and accelerated reproductive aging, accompanied by transient postnatal growth retardation. IGF1R deficiency also altered trabecular and cortical bone structural parameters in both sexes, supporting a role for IGF1R signaling in coordinating growth, skeletal physiology, and reproductive function. Despite reduced food intake and increased energy expenditure in females after adjusting for lean mass, IGF1R deletion caused only modest metabolic alterations, with transient decreases in body weight and largely unchanged body composition and locomotor activity. In contrast, combined deletion of IGF1R and IR in LepRb neurons resulted in marked metabolic disturbances, including increased adiposity, reduced lean mass, lower energy expenditure, decreased locomotor activity, and impaired insulin sensitivity in males. These findings indicate cooperative roles of IGF1R and IR signaling within LepRb neurons in regulating body composition, energy balance, and glucose homeostasis. Together, our results demonstrate that IGF1R signaling in LepRb neurons primarily regulates reproductive development, skeletal physiology, and growth, whereas combined IGF1R and IR signaling is required for maintaining metabolic homeostasis. These findings identify LepRb neurons as an important neuroendocrine hub integrating IGF and insulin signaling to coordinate growth, reproduction, and metabolism in a sex-dependent manner.

## Highlights

• IGF1R signaling in LepRb neurons regulates pubertal timing and fertility.
•IGF1R in LepRb neurons supports normal trabecular and cortical bone mass.
•IGF1R deletion in LepRb neurons causes transient postnatal growth delay.
•Combined IGF1R and IR loss disrupts adiposity and energy expenditure.
•LepRb neurons integrate IGF and insulin signaling across physiology.

## 1 Introduction

Growth and reproduction are tightly interconnected physiological processes that depend on coordinated neuroendocrine and metabolic signaling [1,2]. Insulin-like growth factor 1 (IGF-1) is the major mediator of growth hormone (GH)-stimulated somatic growth, as well as GH-independent anabolic responses such as embryonic growth and reproductive function [3]. IGF-1 administration advances pubertal timing [4]. Notably, ablation of IGF-1 receptors (IGF1R) in the brain causes growth retardation, infertility, and glucose intolerance in mice [5]. To narrow down the neurons through which IGF1R acts, a group of researchers deleted IGF1R in gonadotropin-releasing hormone (GnRH) neurons that control the maturation of the reproductive axis, and only found delayed puberty by 3–4 days with normal adult reproductive function [4]. These findings suggested that other upstream neurons responsive to IGF-1 may alter GnRH neuronal activity and regulate reproductive function.

Neurons expressing the long form of the leptin receptor (LepRb) sense various metabolic cues to regulate multiple physical processes, including puberty onset, adult fertility, energy balance, glucose homeostasis, and bone health [[6], [7], [8], [9], [10], [11], [12], [13], [14], [15], [16], [17], [18]]. Many of the actions of leptin are attributable to effects in LepRb neurons, particularly in the mediobasal hypothalamus, including the arcuate nucleus (ARH) [19,20]. Disruption of ARH LepRb neurons causes modest weight gain [21,22]. LepRb neurons in the dorsomedial hypothalamus co-expressing *Glp1r* suppress food intake and body weight [23], and mediate leptin’s thermoregulatory actions [24]. Unexpectedly, GH signaling in LepRb neurons did not influence body growth or food intake but played a critical role in regulating glucose metabolism [25]. These findings sparked our interest in investigating the role of IGF1R in LepRb neurons in regulating body growth, reproduction, and metabolism.

IGF-1 and insulin act through related tyrosine kinase receptors whose signals converge on downstream insulin receptor substrate (IRS) proteins [26] and then recruit and activate phosphatidylinositol 3-kinase (PI3K) to promote Akt signaling [27]. Among the IRS proteins, IRS2 pathways were found to integrate female reproduction and energy homeostasis, as mice lacking IRS2 displayed small, anovulatory ovaries with decreased numbers of follicles [28]. Loss of IRS2 in LepRb neurons in mice resulted in obesity, glucose intolerance, and insulin resistance, but their reproductive capacity remained normal [12]. PI3K signaling in LepRb neurons plays a crucial role in regulating energy expenditure, reproduction, and body growth [11]. Disruption of PI3K p110α and p110 β subunits increased energy expenditure, locomotor activity, and thermogenesis, while delaying puberty and impairing fertility [11]. Surprisingly, although deletion of IR in LepRb neurons caused a mild delay of puberty, it did not recapitulate the other metabolic and reproductive changes seen in PI3K knockout mice [11]. IGF1R and IR compensate for each other to maintain normal muscle growth [29] and white and brown fat mass formation in mice [30]. Therefore, we hypothesized that the IGF1R and IR in LepRb neurons jointly support metabolic and reproductive function. To test this hypothesis, we generated mice lacking IGF1R exclusively in LepRb neurons (IGF1R^LepRb^ mice) and mice simultaneously lacking both IGF1R and IR in LepRb neurons (IGF1R/IR^LepRb^ mice) and then characterized the impact on the regulation of body growth, reproduction, and metabolism in these models.

## 2 Materials and methods

***Animals and genotyping***. To generate mice with the IGF1Rs specifically deleted in LepRb-expressing neurons, LepR-Cre mice [31] were crossed with IGF1R-floxed mice [32,33] (RRID: IMSR_JAX:012251) and bred to homozygosity for the floxed allele only. The IGF1R^flox/flox^ mice were designed with loxP sites flanking exon 3. Excision of exon 3 in the presence of Cre recombinase results in a frameshift mutation and produces a premature stop codon. Littermates only carrying Cre recombinase were used as controls (LepR-Cre). To generate a double-knockout of IGF1R and IR, LepR-Cre mice [31] were crossed with IGF1R-floxed and IR-floxed mice [34]. For efficient production of experimental animals, breeding colonies were maintained using LepRb-Cre; IR^flox/flox^; IGF1R^flox/+^ or LepRb-Cre; IR^flox/+^; IGF1R^flox/+^ breeders. Control mice consisted of LepRb-Cre-positive animals with intact receptor alleles derived from the same breeding colony. Controls were either littermates or colony-derived non-littermate controls from the same breeding line. All mice were maintained on a C57BL/6 background, housed under identical conditions, and analyzed in parallel. Where specified, the mice also carried the reporter Ai14, in which a loxP-flanked STOP cassette prevents transcription of a CAG promoter-driven red fluorescent protein (tdTomato) inserted into the ROSA26 locus (Jackson Laboratory, stock no.007914) [16,35]. Mice were housed in the University of Toledo College of Medicine animal facility at 22 °C–24 °C on a 12-hour light/12-hour dark cycle and were fed standard rodent chow (2016 Teklad Global 16% Protein Rodent Diet, 12% fat by calories; Harlan Laboratories, Indianapolis, Indiana). On postnatal day (PND) 21, mice were weaned. At the end of the study, all animals were sacrificed by CO_2_ asphyxiation or by cardiac puncture under 2% isoflurane anesthesia to draw blood. Mice were genotyped using the pairs of primers described in Supplemental Table 1. The University of Toledo College of Medicine Institutional Animal Care and Use Committee approved all procedures.

***Puberty and reproductive phenotype assessment***. The timing of pubertal development was checked daily after weaning at 21 days by determining vaginal opening (VO) in female mice and balanopreputial separation (BPS) in male mice. Saline lavages were used to collect vaginal cells of female mice following VO. The first estrus age was identified as two consecutive days with keratinized cells after two previous days with leukocytes [11]. Estrus stages were assessed based on vaginal cell cytology as described previously [11,36]. BPS was checked daily from weaning by manually retracting the prepuce with gentle pressure [37]. After BPS was observed in male mice, each male mouse was paired with one fertile wild-type female to assess the first date of conception, while monitoring daily for copulatory plugs. The paired mice were separated until males reached 8 weeks of age, and pregnancy rate, litter size, and interval from mating to birth were recorded. The age of sexual maturation was estimated by subtracting the average pregnancy duration for mice (21 days) from the birth of the first litter. At 3 months of age, we examined adult fertility. Animals were paired with fertile adult wild-type breeders for 8 nights to collect additional data on pregnancy rates, intervals from mating to birth, and numbers of pups per litter. To examine fertility at different ages, we assessed adult fertility at 4, 7, 10, 14, and 17 months of age. The pregnancy rate and number of pups per litter were recorded accordingly.

***Body length measurement***. Body length, from the tip of the nose to the base of the tail, was measured weekly from week 3–20 when mice were anesthetized under 2% isoflurane.

***Body composition assessment and indirect calorimetry***. Body weight was measured weekly from week 3–20. Body composition was assessed by nuclear magnetic resonance (Minispec mq7.5; Bruker Optics, Billerica, Massachusetts) to determine the percentage of fat mass, lean mass, and body fluid [38]. We performed indirect calorimetry in mice at the age of 3–4 months in a Calorimetry Module (Columbus Instruments, Columbus, Ohio) as described previously [39]. Adult (14- to 16-week-old) IGF1R^LepRb^, IGF1R/IR^LepRb,^ and age-matched control LepRb-Cre (*n* = 7–11/genotype) males and females were weighed and then individually placed into the sealed chambers with free access to food and water. The study was conducted in an experimental room maintained at 21–23 °C with a 12-hour light/dark cycle. The metabolic assessments were conducted continuously for 72 h following a 24-hour adaptation period. The consumption of oxygen (VO_2_) and production of carbon dioxide (VCO_2_) in each chamber were sampled sequentially for 1 min at 20-minute intervals, and motor activity was recorded every second in the x and z dimensions. Respiratory exchange ratio was calculated as VCO_2_/VO_2_, and energy expenditure was calculated based on the formula: EE = 3.941 × VO_2_ + 1.106 × VCO_2_.

***Glucose tolerance test (GTT) and insulin tolerance test (ITT)***. GTTs and ITTs were performed as described previously [38]. For GTTs, after a 16-hour fast, mice were injected with dextrose (2 g/kg i.p.). Tail blood glucose was measured using a veterinary glucometer (AlphaTRAK; Abbott Laboratories, Abbott Park, Illinois) before and 15, 30, 45, 60, 90, and 120 min after injection. For ITT, after a 3-hour fast, mice were injected with recombinant insulin (0.75 U/kg i.p.). Tail blood glucose was measured again at specified time points.

***Hormone assays***. Submandibular blood was collected at 9:00 to 11:00 AM to detect basal luteinizing hormone (LH) and follicle-stimulating hormone (FSH) levels from mice between postnatal day 21 and 28 (before showing vaginal opening or balanopreputial separation) and 3-month-old male and female mice on diestrus. LH and FSH were measured via radioimmunoassay performed by the University of Virginia Center for Research in Reproduction Ligand Assay and Analysis Core (Charlottesville, VA). The assay for LH had a detection sensitivity of 3.28 pg/mL. The intra-assay and inter-assay coefficients of variation (CVs) were 4.0% and 8.6%, respectively. The assay for FSH had a detection sensitivity of 7.62 pg/mL. The intra-assay and inter-assay CVs were 7.4% and 9.1%. Serum IGF-1 was measured by ELISA (Crystal Chem, Elk Grove Village, IL) with a sensitivity of 0.5–18 ng/mL and with intra- and inter-assay CVs <10%. Serum GH was measured by ELISA (Crystal Chem, Elk Grove Village, IL) with a sensitivity range of 0.15–9 ng/mL and intra-assay and inter-assay CVs of <10%. Serum insulin was measured by ELISA (Crystal Chem, Elk Grove Village, IL) with a sensitivity range of 0.1–12.8 ng/mL and intra-assay and inter-assay CVs of <10%. Serum C-Peptide was measured by ELISA (Crystal Chem, Elk Grove Village, IL) with a sensitivity range of 0.37–15 ng/mL and intra-assay and inter-assay CVs of <10%.

***Micro-computed tomography (micro-CT)***. Dissected right femora and lumbar vertebrae from 5-month-old mice (n = 4/genotype) were immersed in 10% formalin and stored in the dark. To determine the tissue microarchitecture and densitometry, bones were scanned using the micro-CT-35 system (Scanco Medical AG, Bruettisellen, Switzerland), as previously described [40]. Scan parameters included 7-micron nominal resolution with the X-ray source operating at 70 kVp, and a current of 113 μA. As described previously [40], scans of the proximal tibia consisted of 300 slices, starting at the growth plate. Images of trabecular bone were segmented at 220 threshold values using a per mille scale following manual contouring, starting 10 slices below the growth plate and extending to the end of the image stack. Scans of cortical bone at the tibia midshaft consisted of 55 slices. Images of cortical bone were contoured in the entire image stack and segmented at 260 thresholds using a per mille scale. The analyses of the trabecular bone microstructure and the cortical bone parameters were performed using Evaluation Program V6.5-1 (Scanco Medical AG, Bruettisellen, Switzerland) and conformed to recommended guidelines [41]. All micro-CT measurements were performed blindly.

***Tissue collection and histology***. Ovaries, testes, white adipose tissue, and brown adipose tissue were collected from mice and fixed immediately in 10% formalin overnight and then transferred to 70% ethanol. Then tissues were embedded in paraffin and cut into 5- to 8-μm sections. Sections were stained with hematoxylin and eosin and then examined under a microscope. For follicle and sperm quantification, a minimum of four ovaries and testes for each genotype at 5-month-old age were collected. Follicles were classified into the following categories: primordial, primary, secondary, and Graafian. Testis sections were analyzed by evaluating sperm stages, including counting the number of spermatogonia, spermatocytes, spermatids, and spermatozoa using light microscopy at 20× magnification [42]. Sperm counts are reported per seminiferous tubule cross-section.

***Quantitative real-time PCR***. Mice were placed under isoflurane anesthesia, followed by decapitation and removal of the hypothalamus. Total hypothalamic RNA was extracted from dissected tissues by an RNeasy Lipid Tissue Mini Kit (QIAGEN, Valencia, California) [43]. Single-strand cDNA was synthesized by a High-Capacity cDNA Reverse Transcription kit (Applied Biosystems) using random hexamers as primers, as listed in Supplemental Table 1. Each sample was analyzed in duplicate to measure gene expression level. qPCR reactions (25 μL) were performed in 96-well plates using SYBR Green qPCR SuperMix/ROX (Smart Bioscience Inc., Maumee, Ohio), gene-specific primers, and 1 μL of cDNA template. The reactions were run in an ABI PRISM 7000 sequence detection system (PE Applied Biosystems, Foster City, California), or a 10 μM cDNA template was used in a 10 μL system in 384-well plates with SYBR Green qPCR SuperMix/ROX (Smart Bioscience Inc., Maumee, Ohio). These reactions were run in a ThermoFisher QuantStudio 5 Real-Time PCR system (Applied Biosystems, Foster City, California). All data were analyzed using the comparative Ct method (2^−ΔΔCt^) with glyceraldehyde-3-phosphate dehydrogenase (GAPDH) as the housekeeping gene. The mRNA expression in IGF1R^LepRb^ and IGF1R/IR^LepRb^ versus LepRb-Cre control mice was determined using a comparative cycle threshold method, and the relative gene copy number was calculated as 2-ΔΔCt, presented as a fold change from the relative mRNA expression of the LepRb-Cre control group.

***Perfusion and BaseScope***. Adult LepRb-Cre, IGF1R^LepRb,^ and IGF1R/IR^LepRb^ mice at the age of 6 months were deeply anesthetized by ketamine and xylazine. After brief perfusion with a saline rinse, mice were perfused transcardially with 10% formalin for 10 min, and the brain was removed. The brain was post-fixed in 10% formalin at 4 °C overnight and then immersed in 10%, 20%, and 30% sucrose solutions at 4 °C for 24 h each. Brains were frozen and sectioned coronally at 20 μm using a cryostat. Sections were washed in DEPC-treated phosphate-buffered saline (PBS) for 10 min, mounted onto DEPC-treated charged slides, air-dried for 30 min at room temperature, and stored at −80 °C until use.

On the day of the BaseScope assay, slides were thawed, rinsed twice in PBS, and incubated at 60 °C for 30 min. Sections were post-fixed in 10% formalin for 15 min at 4 °C, dehydrated through graded ethanol (50%, 70%, and 100%; 5 min each), and subjected to target retrieval for 5 min at 100 °C. Slides were then incubated with Protease III (ACDBio, #322337) for 30 min at 40 °C. After rinsing in distilled water, sections were hybridized with BaseScope probes targeting *Lepr* (ACDBio #842801-C2), *Igf1r* (ACDBio #1889741-C1), or *Insr* (ACDBio #719741-C1) for 2 h at 40 °C according to the manufacturer’s instructions. Signal amplification and chromogenic detection were performed using the BaseScope Duplex Detection Reagent Kit (ACDBio #323810) following the standard BaseScope protocol.

Slides were coverslipped and imaged in bright-field mode using an Olympus Slideview VS200 digital slide scanner. Images were analyzed using ImageJ software. Double-positive cells within the ARH were quantified from three consecutive sections per mouse, and the mean value was used to represent each animal. Three mice per group were included for statistical analyses. Quantification of mRNA colocalization was performed in the ARH only, as the VMH and DMH regions exceeded the field of view for consistent single-image acquisition at the magnification used.

***Statistical analysis***. Data are presented as mean ± SEM. Normality testing was used to determine the normal distribution of data. If the data followed a normal distribution, One-way ANOVA was used as the primary statistical method to compare the three groups, followed by the Tukey multiple comparison test. If the data did not follow a normal distribution, the Kruskal–Wallis test was used. For body weight, body length, GTTs, and ITTs, a two-way ANOVA was used to compare changes over time among three groups. Bonferroni multiple comparison tests were then performed to compare differences between groups. A value of *P* ≤ 0.05 was considered to be significant.

## 3 Results

### 3.1 Disruption of Igf1r expression in LepRb neurons

**Figure 1:**
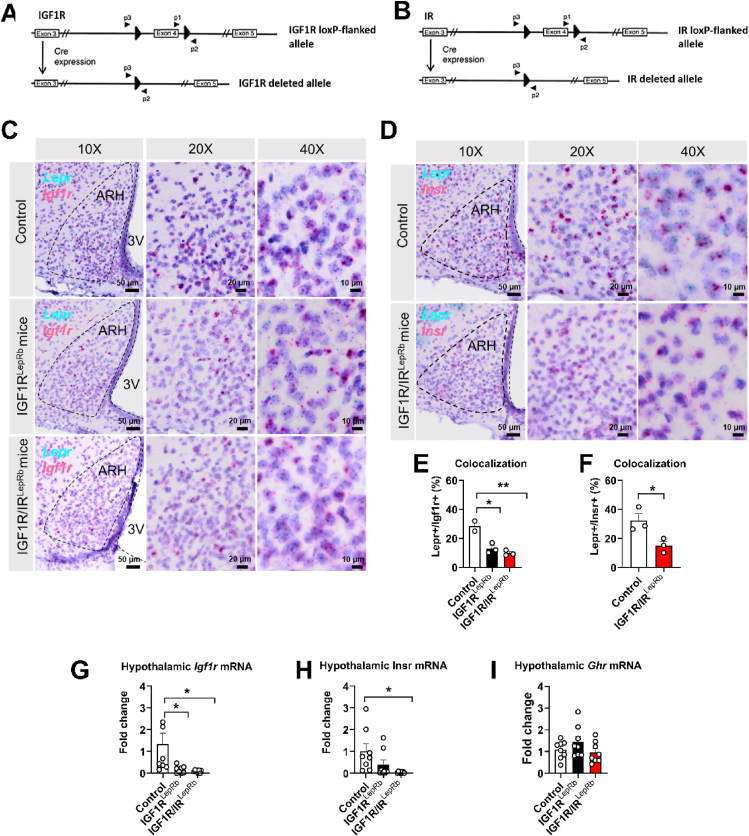
**Generation and validation of LepRb neuron-specific IGF1R and/or IR conditional Knockout mice.** (A–B) Schematic of IGF1R (A) and IR (B) floxed allele and Cre-mediated deletion strategy. (C) BaseScope detection of *Lepr* and *Igf1r* mRNA showing colocalization in control mice and reduced *Igf1r* signal in *Lepr*-expressing neurons of IGF1R^LepRb^ and IGF1R/IR^LepRb^ mice. (D) BaseScope detection of *Lepr* and *Insr* mRNA showing colocalization in control mice and reduced *Insr* signal in *Lepr*-expressing neurons of IGF1R/IR^LepRb^ mice. (E–F) Quantification of *Lepr*-*Igf1r* colocalization (n = 3/group) (E) and *Lepr*-*Insr* colocalization (*n* = 3/group). (G–I) Hypothalamic *Igf1r* (G), *Insr* (H), and *Ghr* (I) mRNA expression measured by quantitative PCR (qPCR) in control, IGF1R^LepRb^, and IGF1R/IR^LepRb^ mice (*n* = 8/group). Values are presented as means ± SEM. Statistical significance was determined by one-way ANOVA followed by Tukey’s post hoc test or two-tailed Student’s *t*-test. ∗*P* < 0.05, ∗∗*P* < 0.01, and ∗∗∗*P* < 0.001.

To generate LepRb neuron-specific conditional knockout mice, LepRb-Cre mice were crossed with mice carrying floxed alleles of *Igf1r* and/or *Insr*, allowing Cre-mediated deletion selectively in LepRb-expressing neurons (Figure 1A–B). Efficient deletion of *Igf1r* transcripts in LepRb neurons was validated using BaseScope in situ hybridization. In control mice, *Igf1r* mRNA was detected in a subset of *Lepr*-expressing neurons within hypothalamic regions including the ARH, ventromedial hypothalamus (VMH), and dorsomedial hypothalamus (DMH) (Figure 1C and Supplemental Figs. 1–2). Quantitative analysis was restricted to the ARH, where anatomical boundaries could be consistently captured within a single imaging field. Approximately 26.8% of *Lepr*-positive neurons co-expressed *Igf1r* mRNA in control mice, whereas colocalization was markedly reduced in IGF1R^LepRb^ mice (13.09%) and further decreased in IGF1R/IR^LepRb^ mice (9.86%), confirming effective deletion of *Igf1r* in LepRb neurons (Figure 1E). Similar qualitative patterns of reduced *Igf1r* colocalization with *Lepr* neurons were observed in the VMH and DMH (Supplementary Fig. 1). Similarly, BaseScope analysis demonstrated robust colocalization of *Insr* with *Lepr* in control mice (33.5%), which was substantially reduced in IGF1R/IR^LepRb^ mice (15.13%) (Figure 1D,F). Consistent qualitative reductions in *Insr* colocalization were also observed in the VMH and DMH (Supplementary Fig. 2).

Consistent with these findings, quantitative PCR analysis of hypothalamic tissue revealed significantly reduced *Igf1r* mRNA expression in IGF1R^LepRb^ and IGF1R/IR^LepRb^ mice, and decreased *Insr* expression in IGF1R/IR^LepRb^ mice, whereas expression of *Ghr* was not altered (Figure 1G–I). Together, these data confirm efficient and selective deletion of IGF1R and/or IR in LepRb neurons.

### 3.2 Delayed puberty and impaired fertility in IGF1RLepRb and IGF1R/IRLepRb mice

**Figure 2:**
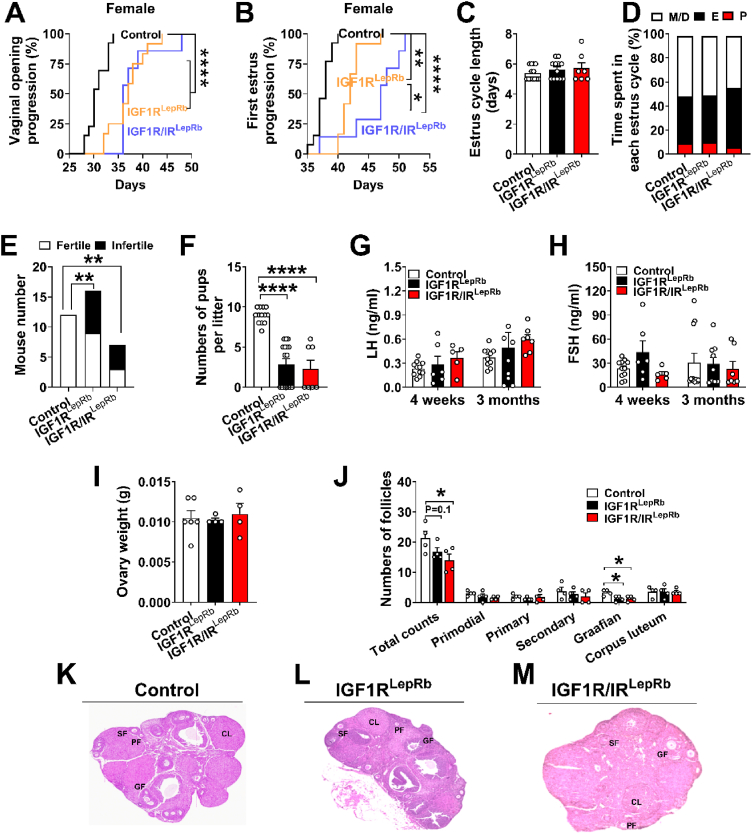
**Reproductive deficits in female IGF1R^LepRb^ and IGF1R/IR^LepRb^ mice.** (A) Vaginal opening age, (B) first estrus age, (C) estrus cycle length, and (D) estrus cyclicity were evaluated in female control, IGF1R^LepRb,^ and IGF1R/IR^LepRb^ mice (*n* = 7–16/group). (E) Pregnancy rate and (F) number of pups per litter in 4-month-old female control, IGF1R^LepRb^ and IGF1R/IR^LepRb^ mice (*n* = 7–16/group). (G–H) Serum levels of LH (G) and FSH (H) on diestrus in 4-week-old and 3-month-old female control, IGF1R^LepRb^ and IGF1R/IR^LepRb^ mice (*n* = 5–11/group). (I) Ovary weight and (J) histological analysis of ovarian follicles (*n* = 4–6/group). (K–M) Representative sections of H&E-stained paraffin-embedded ovaries from 5-month-old female control, IGF1R^LepRb^ and IGF1R/IR^LepRb^ mice. PF, primary follicle; SF, secondary follicle; GF, Graafian follicle; CL, corpus luteum. E, estrus; M/D, metestrus/diestrus; P, proestrus. Values are presented as means ± SEM. Statistical significance was determined by Log Rank Test or Chi-square or one-way ANOVA followed by Tukey’s post hoc test. ∗*P* < 0.05, ∗∗*P* < 0.01, ∗∗∗*P* < 0.001, ∗∗∗∗*P* < 0.0001.

Female IGF1R^LepRb^ mice exhibited delayed pubertal onset, as evidenced by significantly later vaginal opening (at 37.0 ± 1.0 days in IGF1R^LepRb^ vs 30.8 ± 0.5 days in controls) and first estrus age (at 42.1 ± 0.5 days in IGF1R^LepRb^ vs 37.6 ± 0.4 days in controls) (Figure 2A–B). Despite this delay, estrous cycle length and time spent in each estrous stage were comparable to controls (Figure 2C–D). At 3 months of age, female IGF1R^LepRb^ mice displayed impaired fertility, characterized by reduced pregnancy rates and fewer pups per litter (Figure 2E–F). Circulating LH and FSH levels were not significantly altered (Figure 2G–H), and ovary weights were similar between groups (Figure 2I). However, histological analysis revealed fewer Graafian follicles in 5-month-old IGF1R^LepRb^ females (Figure 2J–M), suggesting compromised follicular maturation that may contribute to the observed fertility deficits.

Female IGF1R/IR^LepRb^ mice showed reproductive phenotypes broadly similar to IGF1R^LepRb^ mice, although the delay in first estrus was more pronounced (Figure 2A–J), suggesting that IR signaling in LepRb neurons may also contribute to pubertal timing.

**Figure 3:**
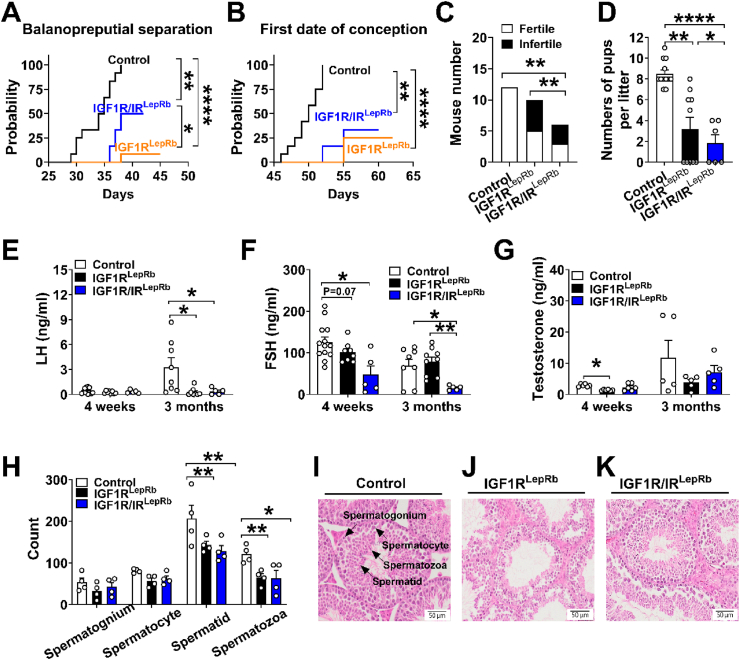
**Reproductive deficits in male IGF1R^LepRb^ and IGF1R/IR^LepRb^ mice.** (A) Balanopreputial separation age and (B) first date of conception in male control, IGF1R^LepRb^, and IGF1R/IR^LepRb^ mice (*n* = 6–12/group). (C) Pregnancy rate and (D) numbers of pups per litter in male control, IGF1R^LepRb^, IGF1R/IR^LepRb^ mice (*n* = 6–12/group). (E) Serum LH, (F) FSH, and (G) testosterone levels in 4-week-old and 3-month-old male control, IGF1R^LepRb^ and IGF1R/IR^LepRb^ mice (*n* = 5–12/group). (H) Analysis of sperm developmental stages within seminiferous tubules (*n* = 4/group) and (I–K) representative sections of H&E-stained paraffin-embedded testes in 5-month-old male control, IGF1R^LepRb^ and IGF1R/IR^LepRb^ mice. Values are presented as means ± SEM. Statistical significance was determined by Log Rank Test or Chi-square or one-way ANOVA followed by Tukey’s post hoc test. ∗*P* < 0.05, ∗∗*P* < 0.01, ∗∗∗*P* < 0.001.

In males, IGF1R^LepRb^ mice exhibited delayed balanopreputial separation (41.5 ± 0.6 days of age in IGF1R^LepRb^ vs 33.8 ± 0.9 days in controls) and delayed first successful mating (57.4 ± 1.1 days of age in IGF1R^LepRb^ vs 49.8 ± 0.9 days in controls) (Figure 3A–B). At 3 months, these mice displayed reduced fertility and fewer pups per litter (Figure 3C–D). Hormonal analysis revealed decreased LH levels at 3 months and reduced testosterone levels at 4 weeks (Figure 3E–G). Testicular histology showed reduced numbers of spermatids and spermatozoa within seminiferous tubules of 5-month-old IGF1R^LepRb^ males (Figure 3H–K), consistent with impaired spermatogenesis.

Male IGF1R/IR^LepRb^ mice exhibited more pronounced reproductive deficits than IGF1R^LepRb^ mice, including greater reductions in litter size, lower LH levels at 3 months of age, and reduced FSH levels at both 4 weeks and 3 months (Figure 3E–G). Together, these findings indicate that IGF1R signaling in LepRb neurons plays a critical role in reproductive maturation and fertility in both sexes, with IR signaling providing additional modulatory effects.

**Figure 4:**
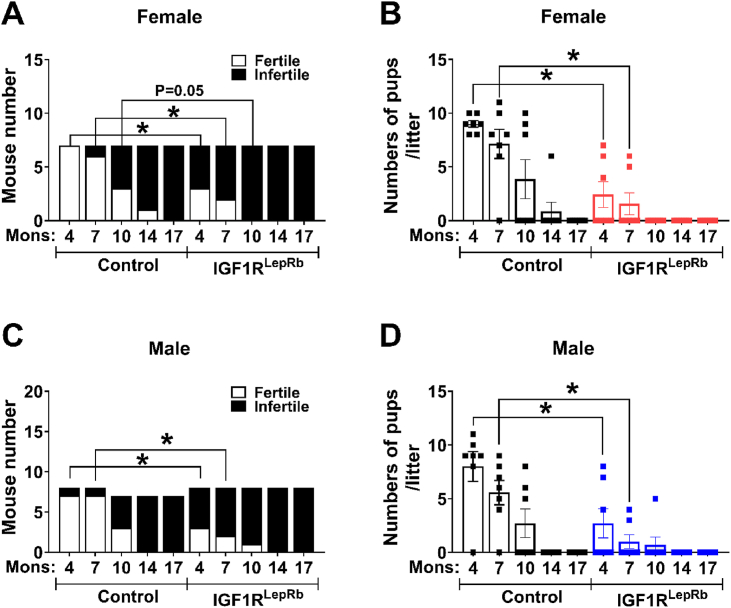
**Advanced reproductive aging in IGF1R^LepRb^ mice.** (A) Pregnancy rate and (B) litter size were measured in 7-, 10-, 14-, and 17-month-old female control and IGF1R^LepRb^ mice (*n* = 7/group). (C) Pregnancy rate and (D) litter size in 7-, 10-, 14-, and 17-month-old male control and IGF1R^LepRb^ mice (*n* = 7/group). Values are presented as means ± SEM. Statistical significance was determined by two-tailed Student’s *t*-test. ∗*P* < 0.05.

A recent study reported that *Igf1* gene therapy induces GnRH release in the median eminence and maintains kisspeptin production in middle-aged female rats [44], suggesting IGF-1 may have a protective effect against reproductive decline. To examine whether IGF1R signaling in LepRb neurons influences reproductive aging, fertility was assessed longitudinally in IGF1R^LepRb^ and control mice at 4, 7, 10, 14, and 17 months of age. At 4 months, female IGF1R^LepRb^ mice already exhibited reduced fertility and smaller litter sizes (Figure 4A–B). Fertility declined more rapidly with age in IGF1R^LepRb^ females, reaching complete infertility by 10 months, whereas control females retained approximately 50% fertility at this age (Figure 4A). Similarly, male IGF1R^LepRb^ mice exhibited accelerated age-related fertility decline compared with controls (Figure 4C–D). These findings suggest that IGF1R signaling in LepRb neurons contributes to maintenance of reproductive function during aging.

### 3.3 Body growth in IGF1RLepRb and IGF1R/IRLepRb mice and bone phenotype of IGF1RLepRb mice

**Figure 5:**
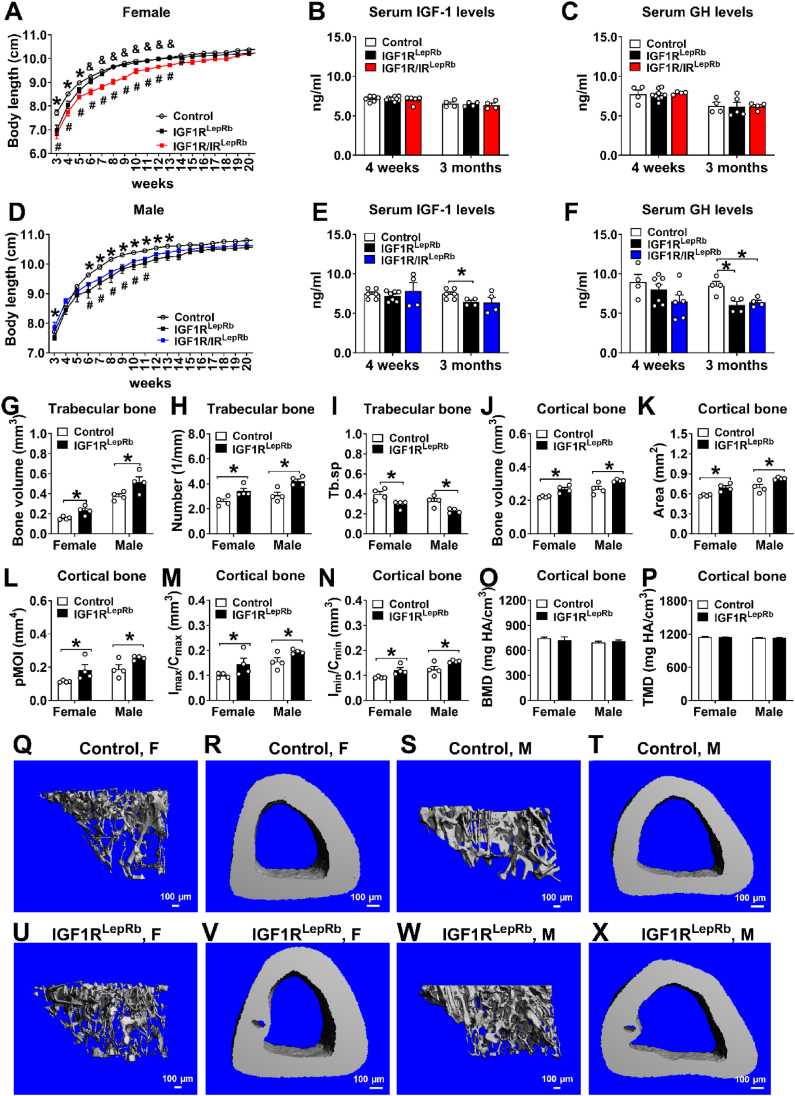
**Body growth and bone phenotype in mice.** (A) Body length curves from week 3–20 in female control, IGF1R^LepRb^, IGF1R/IR^LepRb^ mice (*n* = 7–13/group). (B) Serum levels of IGF-1 and (C) GH at 4 weeks and 3 months of age in female control, IGF1R^LepRb^, and IGF1R/IR^LepRb^ mice (*n* = 4–9/group). (D) Body length curves from week 3–20 in male control, IGF1R^LepRb^, IGF1R/IR^LepRb^ mice (*n* = 7–13/group). (E) Serum levels of IGF-1 and (F) GH at 4 weeks and 3 months of age in male control female control, IGF1R^LepRb^, and IGF1R/IR^LepRb^ mice (*n* = 4–7/group). (G) Trabecular bone volume, (H) trabecular number (Tb.N), (I) spacing (Tb.sp) and (J) cortical bone volume, (K) area (B.Ar), (L) polar moment of inertia (pMOI), (M) resistance to bending across the maximal (I_max_/C_max_), (N) minimal centroid edge (I_min_/C_min_), (O) bone mineral density (BMD) and (P) tissue mineral density (TMD) in female and male control and IGF1R^LepRb^ mice (*n* = 4/group). (Q–X) Representative images of trabecular and cortical bone in female and male control and IGF1R^LepRb^ mice. Values are presented as means ± SEM. Statistical significance was determined by two-tailed Student’s *t*-test, one-way ANOVA followed by Tukey’s post hoc test or two-way ANOVA followed by Bonferroni’s multiple comparison test. ∗*P* < 0.05 (control vs IGF1R^LepRb^ mice), ^#^*P* < 0.05 (control vs IGF1R/IR^LepRb^ mice), ^&^*P* < 0.05 (IGF1R^LepRb^ vs IGF1R/IR^LepRb^ mice).

To determine whether IGF1R signaling in LepRb neurons influences body growth and skeletal health, we assessed longitudinal body growth, circulating hormonal markers, and bone structure using micro-computed tomography (micro-CT). Female IGF1R^LepRb^ mice exhibited transient growth retardation between 3 and 6 weeks of age (Figure 5A) but circulating IGF-1 and GH levels at 4 weeks and 3 months were comparable to controls (Figure 5B–C). Female IGF1R/IR^LepRb^ mice showed a more pronounced yet similarly transient reduction in body growth relative to IGF1R^LepRb^ and control mice, without significant changes in circulating IGF-1 or GH compared to controls (Figure 5A–C).

In males, IGF1R^LepRb^ and IGF1R/IR^LepRb^ mice exhibited transient growth retardation (Figure 5D). Unlike females, this was associated with reduced circulating IGF-1 and GH levels at 3 months of age (Figure 5E–F), suggesting sex-specific hormonal regulation of growth downstream of IGF1R signaling in LepRb neurons. Notably, additional IR deletion did not markedly exacerbate growth or hormonal phenotypes beyond those observed in IGF1R^LepRb^ mice, indicating a predominant role for IGF1R signaling in this context.

We next examined bone phenotype in 5-month-old IGF1R^LepRb^ mice using micro-computed tomography (micro-CT). Compared to controls, female and male IGF1R^LepRb^ mice both displayed significantly increased trabecular bone volume, number, and spacing (Tb.sp) (Figure 5G–I) and cortical bone volume, area, polar moment of inertia, resistance to bending across the maximal (I_max_/C_max_), and minimal centroid edge (I_min_/C_min_) (Figure 5G–N). These skeletal changes were comparable between sexes, indicating minimal sexual dimorphism. This contrasts with prior reports of sex-specific effects of LepRb signaling on bone metabolism, suggesting that IGF1R signaling in LepRb neurons may influence skeletal integrity through a largely sex-independent mechanism [45].

Despite these structural changes, bone mineral density (BMD) or tissue mineral density (TMD) were not significantly altered in either sex (Figure 5O–P). Representative micro-CT renderings of trabecular and cortical bone are shown in Figure 5Q–X. Together, these findings indicate that IGF1R signaling in LepRb neurons contributes to normal postnatal growth and skeletal structure. Bone phenotypes were not assessed in IGF1R/IR^LepRb^ mice. These findings suggest a neuroendocrine mechanism linking metabolic sensing in LepRb neurons to skeletal homeostasis.

### 3.4 Assessment of energy homeostasis in IGF1RLepRb and IGF1R/IRLepRb mice

**Figure 6:**
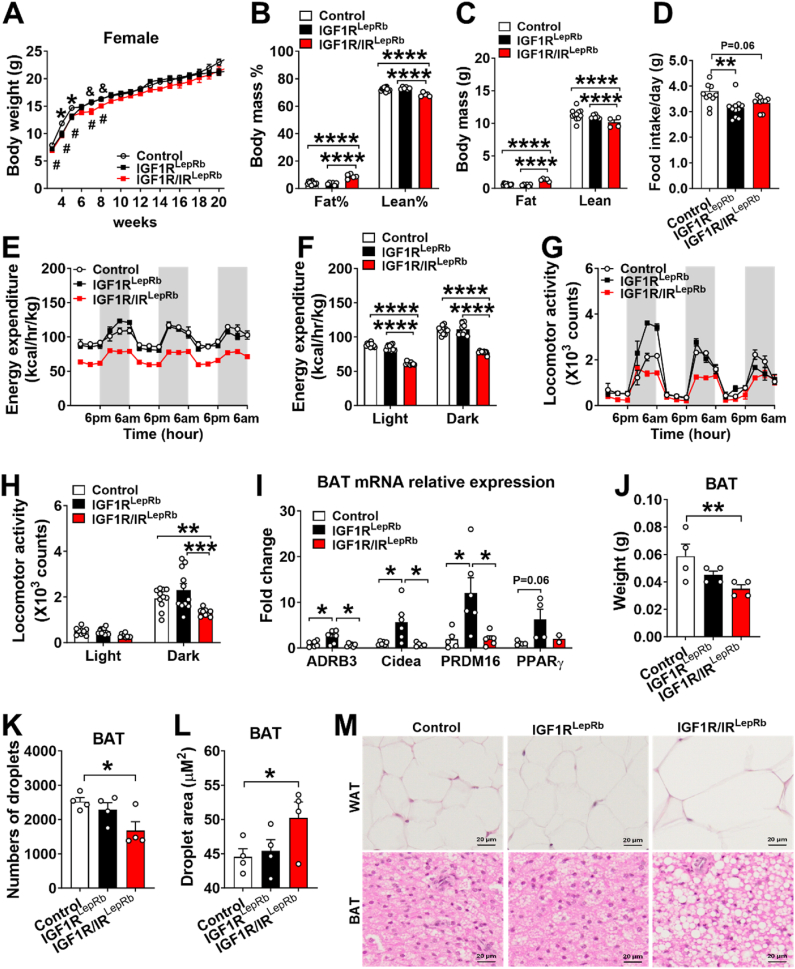
**Altered energy homeostasis in female IGF1R^LepRb^ and IGF1R/IR^LepRb^ mice.** (A) Body weight curves from week 3–20 in female control, IGF1R^LepRb,^ and IGF1R/IR^LepRb^ mice (n = 7–13/group). (B) Body fat mass percentage, (C) lean mass percentage, and (D) food intake in 2-month-old female control, IGF1R^LepRb^ and IGF1R/IR^LepRb^ mice (*n* = 5–11/group). (E–F) Energy expenditure and (G–H) physical activity in 3-month-old female control, IGF1R^LepRb^ and IGF1R/IR^LepRb^ mice (*n* = 9–11/group). (I) Relative expression of thermogenesis markers as measured by quantitative PCR in brown adipose tissue (BAT) and (J) BAT weight in 5-month-old female control, IGF1R^LepRb,^ and IGF1R/IR^LepRb^ mice (*n* = 4–6/group). (K) Number of droplets and (L) droplet area in BAT (*n* = 4/group) and sections of H&E-stained paraffin-embedded BAT (M–O) in 5-month-old female control, IGF1R^LepRb^ and IGF1R/IR^LepRb^ mice. Values are presented as means ± SEM. Statistical significance was determined by one-way ANOVA followed by Tukey’s post hoc test or two-way ANOVA followed by Bonferroni’s multiple comparison test. ∗*P* < 0.05, ∗∗*P* < 0.01, ∗∗∗*P* < 0.0001, and ∗∗∗∗*P* < 0.00001. Symbols denote group comparisons: ∗*P* < 0.05 (control vs IGF1R^LepRb^ mice), ^#^*P* < 0.05 (control vs IGF1R/IR^LepRb^ mice), ^&^*P* < 0.05 (IGF1R^LepRb^ vs IGF1R/IR^LepRb^ mice).

We next evaluated the metabolic phenotype in IGF1R^LepRb^ and IGF1R/IR^LepRb^ mice. Female IGF1R^LepRb^ mice exhibited a transient reduction in body weight without significant changes in body composition (Figure 6A–C). Food intake was significantly reduced compared with controls (Figure 6D). Energy expenditure was significantly increased (Figure 6E–F), and this difference remained significant after adjustment for lean mass in female IGF1R^LepRb^ (Supplemental Fig. 3A). Despite reduced food intake and increased energy expenditure, overall body weight remained largely unchanged, suggesting that additional mechanisms, such as compensatory changes in nutrient utilization or energy partitioning, may contribute to maintaining overall energy balance. No significant changes were detected in locomotor activity during the 72-h measurements (Figure 6G–H). No overt morphological changes in brown adipose tissue (BAT), including BAT weight, lipid droplet number, droplet area, or histology, were detected (Figure 6J–M). Notably, expression of several thermogenesis-related genes in BAT, including *Adrb3*, *Cidea,* and *Prdm16*, was increased (Figure 6I), suggesting modest activation of thermogenic pathways that may contribute to the elevated energy expenditure observed after adjustment for lean mass.

In contrast, female IGF1R/IR^LepRb^ mice displayed markedly increased absolute and relative fat mass and decreased lean mass compared with controls (Figure 6B–C), accompanied by a trend toward reduced food intake (Figure 6D). Energy expenditure was increased after adjustment for lean mass, whereas locomotor activity was decreased (Figure 6E–H and Supplemental Fig. 3A). These changes were associated with decreased BAT weight and increased lipid droplet size, consistent with BAT whitening (Figure 6J–M). Together, these findings indicate that combined deletion of IGF1R and IR in LepRb neurons plays a significant role in regulating body composition and energy expenditure.

**Figure 7:**
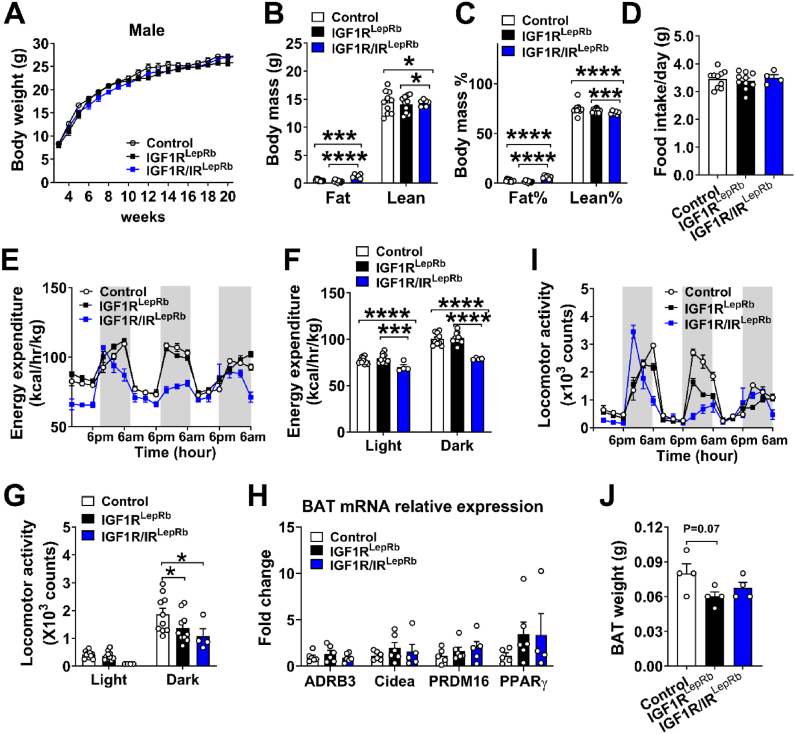
**Altered energy homeostasis in male IGF1R^LepRb^ and IGF1R/IR^LepRb^ mice.** (A) Body weight curves from week 3–20 in male control, IGF1R^LepRb,^ and IGF1R/IR^LepRb^ mice (*n* = 6–10/group). (B) Body fat mass percentage, (C) lean mass percentage, and (D) food intake in 2-month-old male control, IGF1R^LepRb^ and IGF1R/IR^LepRb^ mice (*n* = 4–10/group). (E–F) Energy expenditure and (G–H) physical activity in 3-month-old male control, IGF1R^LepRb^ and IGF1R/IR^LepRb^ mice (*n* = 4–10/group). (I) Relative expression of thermogenesis markers as measured by quantitative PCR in brown adipose tissue (BAT) and (J) BAT weight in 5-month-old male control, IGF1R^LepRb^ and IGF1R/IR^LepRb^ mice (*n* = 4–6/group). Values are presented as means ± SEM. Statistical significance was determined by one-way ANOVA followed by Tukey’s post hoc test or two-way ANOVA followed by Bonferroni’s multiple comparison test. ∗*P* < 0.05, ∗∗*P* < 0.01, ∗∗∗*P* < 0.001, and ∗∗∗∗*P* < 0.0001.

In males, IGF1R^LepRb^ mice showed no significant differences in body weight, body composition, or food intake compared to controls (Figure 7A–D). Similar to females, loss of IGF1R was associated with increased energy expenditure and decreased locomotor activity (Figure 7E–G). However, after adjustment for lean mass using ANCOVA, energy expenditure was no longer significantly different from controls (Supplemental Fig. 3B), suggesting that the apparent difference in energy expenditure was largely explained by differences in body composition. Consistent with these findings, no significant differences were observed in thermogenic gene expression or BAT weight compared with controls (Figure 7H–J). In contrast, male IGF1R/IR^LepRb^ mice exhibited increased fat mass, reduced lean mass, increased energy expenditure and decreased locomotor activity (Figure 7B–G). However, this increased energy expenditure was no longer significant after adjustment for lean mass (Supplemental Fig. 3B). Together, these findings support the cooperative role of IGF1R and IR signaling in LepRb neurons in maintaining metabolic homeostasis.

### 3.5 Assessment of glucose homeostasis in IGF1RLepRb and IGF1R/IRLepRb mice

To determine whether loss of IGF1R and/or IR in LepRb neurons affects glucose homeostasis, we evaluated glucose tolerance, insulin sensitivity, and related metabolic parameters in IGF1R^LepRb^ and IGF1R/IR^LepRb^ mice. Female IGF1R^LepRb^ mice exhibited modest glucose intolerance at 30 min and 45 min during the GTT, although the overall area under the curve (AUC) was not significantly changed (Supplemental Fig. 4A–B). Insulin tolerance tests (ITT) revealed no significant differences compared with controls (Supplemental Fig. 4C–D). Interestingly, fasting glucose levels were modestly reduced in female IGF1R^LepRb^ mice despite unchanged circulating insulin and C-peptide levels (Supplemental Fig. 4E–G). Consistent with these findings, hepatic expression of the gluconeogenic enzyme glucose-6-phosphatase (*G6pc*) was significantly decreased (Supplemental Fig. 4H), suggesting a potential reduction in hepatic gluconeogenic output.

In contrast, female IGF1R/IR^LepRb^ mice did not show significant changes in glucose homeostasis, circulating insulin or C-peptide levels, or hepatic gene expression (Supplemental Fig. 4A–H, N–O). These findings indicate that deletion of IGF1R alone produces modest alterations in glucose metabolism, whereas combined deletion of IGF1R and IR in LepRb neurons does not significantly alter glucose homeostasis in female mice.

Male IGF1R^LepRb^ mice did not display glucose intolerance or insulin resistance (Supplemental Fig. 4I–L). However, unlike females, male IGF1R^LepRb^ mice exhibited elevated fasting glucose levels accompanied by increased hepatic expression of gluconeogenic genes (*G6pc* and *Pepck*) (Supplemental Fig. 4M and P), suggesting a potential increase in hepatic glucose production. Insulin resistance was observed only in male IGF1R/IR^LepRb^ mice (Supplemental Fig. 4K–L), supporting a cooperative role of IGF1R and IR signaling in LepRb neurons in maintaining insulin sensitivity. Notably, the elevated fasting glucose levels and gluconeogenic gene expression observed in male IGF1R^LepRb^ mice were not detected in male IGF1R/IR^LepRb^ mice (Supplemental Fig. 4M and P). Collectively, these findings indicate that IGF1R and IR signaling in LepRb neurons contribute to the regulation of glucose homeostasis in a sex-dependent manner.

## 4 Discussion

This study identifies IGF1R signaling in LepRb-expressing neurons as an important regulator of reproductive maturation, somatic growth, skeletal homeostasis, energy balance, and glucose metabolism (Supplemental Table 2). Our previous work showed that genetic ablation of IR alone in LepRb neurons results in a mild delay in puberty [11]. Extending these findings, the present study indicates that IGF1R and IR signaling interact within LepRb neurons to regulate reproductive function, body composition, energy expenditure, and insulin sensitivity. Notably, IGF1R deletion alone primarily affected reproductive development, fertility, skeletal phenotypes, and aspects of energy homeostasis, whereas combined IGF1R/IR deletion produced more pronounced metabolic alterations, including increased adiposity, reduced lean mass and impaired insulin sensitivity. These findings suggest that LepRb neurons integrate metabolic and reproductive signals through partially overlapping IGF1R- and IR-dependent pathways.

IGF1R signaling in the brain has well-established roles in development of the somatotropic axis, peripheral growth, and metabolic regulation [5]. Homozygous brain-specific IGF1R knockout mice exhibit microcephaly, severe growth retardation, infertility, and paradoxically elevated circulating IGF-1 levels, whereas heterozygous mutants develop progressive growth retardation beginning around three weeks of age [5]. These findings suggest compensatory endocrine feedback within the somatotropic axis. Hypophysiotropic somatostatin neurons can sense circulating IGF-1 [46], yet selective ablation of IGF1R or GHR in these neurons does not significantly alter body growth or circulating IGF-1 levels [47], indicating redundancy in central growth regulatory circuits. Similarly, deletion of IGF1R in kisspeptin neurons produces persistent growth retardation [48]. Together, these observations support a model in which multiple hypothalamic neuronal populations cooperatively regulate somatic growth. The relatively modest growth phenotypes observed after LepRb-specific IGF1R deletion likely reflect both cell-type specificity and post-developmental timing of receptor loss.

Consistent with this framework, both IGF1R^LepRb^ and IGF1R/IR^LepRb^ mice exhibited transient growth retardation. Reduced circulating IGF-1 and GH levels were observed in male mice at three months of age but not in females, suggesting sex-specific neuroendocrine regulation downstream of LepRb neuronal IGF1R signaling. Previous studies disrupting PI3K signaling downstream of LepRb also reported growth retardation despite normal circulating IGF-1 levels [11], possibly reflecting the limitations of single time-point hormone measurements given pulsatile GH secretion. Interestingly, IGF1R deletion in LepRb neurons increased trabecular bone volume, cortical bone area, and indices of bone strength, contrasting with skeletal phenotypes reported following disruption of PI3K signaling downstream of LepRb [11]. This discrepancy may reflect developmental timing or pathway-specific signaling effects. Delayed pubertal onset in IGF1R^LepRb^ mice could shift the trajectory of bone accrual, potentially prolonging the period of peak bone mass development. Similar phenomena have been described clinically in individuals with delayed puberty, although the relationship between pubertal timing and bone turnover remains debated [[49], [50], [51]]. Further studies are needed to clarify mechanisms underlying the altered skeletal phenotype.

The interaction between central IGF1R signaling and local skeletal regulation is also an important consideration. Skeletal stem cells express LepRb and IGF1R and locally regulate bone formation and marrow adiposity [45]; [[52], [53], [54]]. Although direct evidence for co-expression within the same cell population remains limited, such overlap is plausible. Notably, inhibition of IGF-1 signaling in bone typically impairs bone growth [[54], [55], [56]], whereas IGF1R deletion in LepRb neurons increased bone parameters in our model. This suggests that central IGF1R signaling may exert dominant regulatory effects on skeletal homeostasis or indirectly influence bone via neuroendocrine pathways such as delayed puberty. Further studies dissecting central versus peripheral IGF1R signaling will be important.

In addition to skeletal phenotypes, IGF1R deletion in LepRb neurons produced clear reproductive alterations, including delayed pubertal onset, reduced fertility, and accelerated reproductive aging. These findings suggest that IGF1R signaling in LepRb neurons contributes to neuroendocrine pathways that coordinate reproductive function with metabolic status. The ARH contains neuronal populations that play key roles in integrating reproductive and metabolic signals [[57], [58], [59]]. LepRb and Kiss1 neuronal populations partially overlap but remain largely distinct [60]. Similar phenotypes following IGF1R deletion in these populations may therefore reflect convergence on shared metabolic–reproductive circuits rather than complete cellular overlap. Comparative analyses suggest overlapping roles in regulating pubertal onset, fertility, and growth, while combined IGF1R/IR deletion additionally affects body composition and male glucose homeostasis. These observations suggest that IGF1R signaling in LepRb neurons may exert broader systemic effects, potentially reflecting the central role of leptin signaling in coordinating body growth, energy balance and reproduction.

Leptin strongly modulates hypothalamic melanocortin circuits, stimulating anorexigenic POMC neurons while inhibiting AgRP neurons [61]. POMC neurons project to reproductive neuroendocrine circuits and can influence GnRH neurons [62], thereby linking metabolic status with reproductive function. Female IGF1R^LepRb^ mice exhibited reduced food intake and increased energy expenditure, accompanied by elevated expression of thermogenic genes in brown adipose tissue. These changes may be consistent with increased melanocortin signaling, although IGF1R expression specifically within POMC neurons was not examined in the present study. Future work will be required to determine whether IGF1R signaling in LepRb neurons modulates metabolic–reproductive coupling through melanocortin circuits.

Recent work identified a brain-bone endocrine axis in which cellular communication network factor 3 (CCN3) secreted from ARH Kiss1 neurons promotes skeletal stem cell activity and bone remodeling [63]. CCN3 can cooperate with IGF-1 signaling to support musculoskeletal cell proliferation [64]. Our findings that IGF1R deletion in LepRb neurons alters both bone structure and reproductive function raise the possibility that IGF1R signaling in LepRb neurons may interact with this emerging brain-bone axis, although direct mechanisms remain to be determined.

Reproductive function declines with age [65,66], and pharmacologic inhibition of IGF1R signaling has been linked to lifespan extension in female mice [64]. Our data suggests that IGF1R signaling in LepRb neurons may contribute to maintaining reproductive function during aging in both sexes. Further work will be required to define how central IGF1R signaling influences ovarian and testicular aging.

Combined IGF1R/IR deletion produced markedly increased fat mass, reduced lean mass, and impaired insulin sensitivity, particularly in males. Peripheral studies show cooperative IGF1R–IR actions in adipose tissue and muscle [29,30], whereas central manipulations often yield divergent metabolic outcomes. For example, adipose-specific IGF1R/IR double knockout mice show reduced fat mass [29,30], while neuronal IR knockout mice develop diet-sensitive obesity [67]. Consistent with these observations, concurrent IGF1R and IR deletion in LepRb neurons produced stronger metabolic effects than IGF1R deletion alone, supporting cooperative signaling within central metabolic circuits. Importantly, IGF1R deletion alone did not significantly alter fat mass, indicating that adiposity changes primarily reflect combined loss of IGF1R and IR signaling. LepRb neurons therefore represent an important neural site integrating insulin and IGF-1 signaling to coordinate metabolic and reproductive endocrine regulation. Future studies under obesogenic conditions such as high-fat diet exposure will help clarify how these pathways contribute to metabolic adaptation.

In summary, IGF1R and IR signaling in LepRb neurons play complementary roles in coordinating growth, skeletal physiology, reproduction, energy balance, body composition, and glucose metabolism. Deletion of IGF1R alone primarily affected growth, skeletal phenotypes, reproduction, and components of energy balance, whereas combined deletion of IGF1R and IR produced more pronounced metabolic alterations, including increased adiposity and impaired insulin sensitivity. These findings identify LepRb neurons as an important neural site where IGF-1 and insulin signaling converge to integrate metabolic and reproductive endocrine regulation.

## CRediT authorship contribution statement

**Mengjie Wang:** Writing – review & editing, Writing – original draft, Investigation, Formal analysis, Data curation. **Piotr J. Czernik:** Data curation. **Beata Lecka-Czernik:** Formal analysis. **Jennifer W. Hill:** Writing – review & editing, Supervision, Funding acquisition, Formal analysis, Conceptualization.

## Declaration of competing interest

The authors declare that they have no known competing financial interests or personal relationships that could have appeared to influence the work reported in this paper.

## Data Availability

Data will be made available on request.
