# Supplementary material for: IGF-1 and insulin receptors in LepRb neurons jointly regulate body growth, bone mass, reproduction, and metabolism
Source: Mol Metab. 2026 Mar 16;107:102355. doi: 10.1016/j.molmet.2026.102355 (PMC13054536; doi:10.1016/j.molmet.2026.102355)
Supplement: Multimedia component 1 [file mmc1.docx]

**Supplementary Figure legends**

**Supplemental Figure 1. Representative BaseScope detection of *Lepr* and *Igf1r* mRNA colocalization in the VMH and DMH.**

(A–B) BaseScope in situ hybridization detecting *Lepr* and *Igf1r* mRNA in hypothalamic sections. Colocalization of *Lepr* and *Igf1r* mRNA is observed in control mice, whereas *Igf1r* signal is reduced in *Lepr*-expressing neurons of IGF1R^LepRb^ and IGF1R/IR^LepRb^ mice in the ventromedial hypothalamus (VMH; A) and dorsomedial hypothalamus (DMH; B). VMH, ventromedial hypothalamus; DMH, dorsomedial hypothalamus.

**Supplemental Figure 2. Representative BaseScope detection of Lepr and Insr mRNA colocalization in the VMH and DMH.**
(A–B) BaseScope in situ hybridization detecting *Lepr* and *Insr* mRNA in hypothalamic sections. Colocalization of *Lepr* and *Insr* mRNA is observed in control mice, whereas *Igf1r* signal is reduced in *Lepr*-expressing neurons of IGF1R/IR^LepRb^ mice in the ventromedial hypothalamus (VMH; A) and dorsomedial hypothalamus (DMH; B). VMH, ventromedial hypothalamus; DMH, dorsomedial hypothalamus.

**Supplemental Figure 3. ANCOVA analysis of energy expenditure adjusted by lean mass.**

(A-B) Energy expenditure was analyzed by ANCOVA with body weight as a covariate to assess genotype effects independent of differences in lean mass in female (A) and male (B) mice. Data are presented as mean ± SEM.

**Supplemental Figure 4. Changes in glucose homeostasis in IGF1R^LepRb^ and IGF1R/IR^LepRb^ mice**. (A) Glucose tolerance test (GTT), (B) area under the curve of GTT (GTT-AUC), (C) insulin tolerance test (ITT), and (D) AUC of ITT (ITT-AUC) of 3-month-old female control, IGF1R^LepRb^ and IGF1R/IR^LepRb^ mice (n=9-14/group). (E) Fasting glucose, (F) insulin, (G) and C-peptide levels in 3-month-old female control, IGF1R^LepRb,^ and IGF1R/IR^LepRb^ mice (n=5-10/group). (H) Relative expression of gluconeogenesis and inflammatory markers in the liver as measured by quantitative PCR in 5-month-old female control, IGF1R^LepRb,^ and IGF1R/IR^LepRb^ mice (n=8/group). (I) GTT, (J) GTT-AUC, (K) ITT, and (L) ITT-AUC of 3-month-old male control, IGF1R^LepRb^ and IGF1R/IR^LepRb^ mice (n=6-10/group). (M) Fasting glucose, (N) insulin, (O) C-peptide levels in 3-month-old male control, IGF1R^LepRb,^ and IGF1R/IR^LepRb^ mice (n=5-6/group). (P) Relative expression of gluconeogenesis markers in the liver as measured by quantitative PCR in 5-month-old male control, IGF1R^LepRb^ and IGF1R/IR^LepRb^ mice (n=6/group). Values are presented as means ±SEM. Statistical significance was determined by one-way ANOVA followed by Tukey’s post hoc test or two-way ANOVA followed by Bonferroni’s multiple comparison test. **P* < 0.05, ***P* < 0.01, ****P* < 0.001, and *****P* < 0.0001.

**
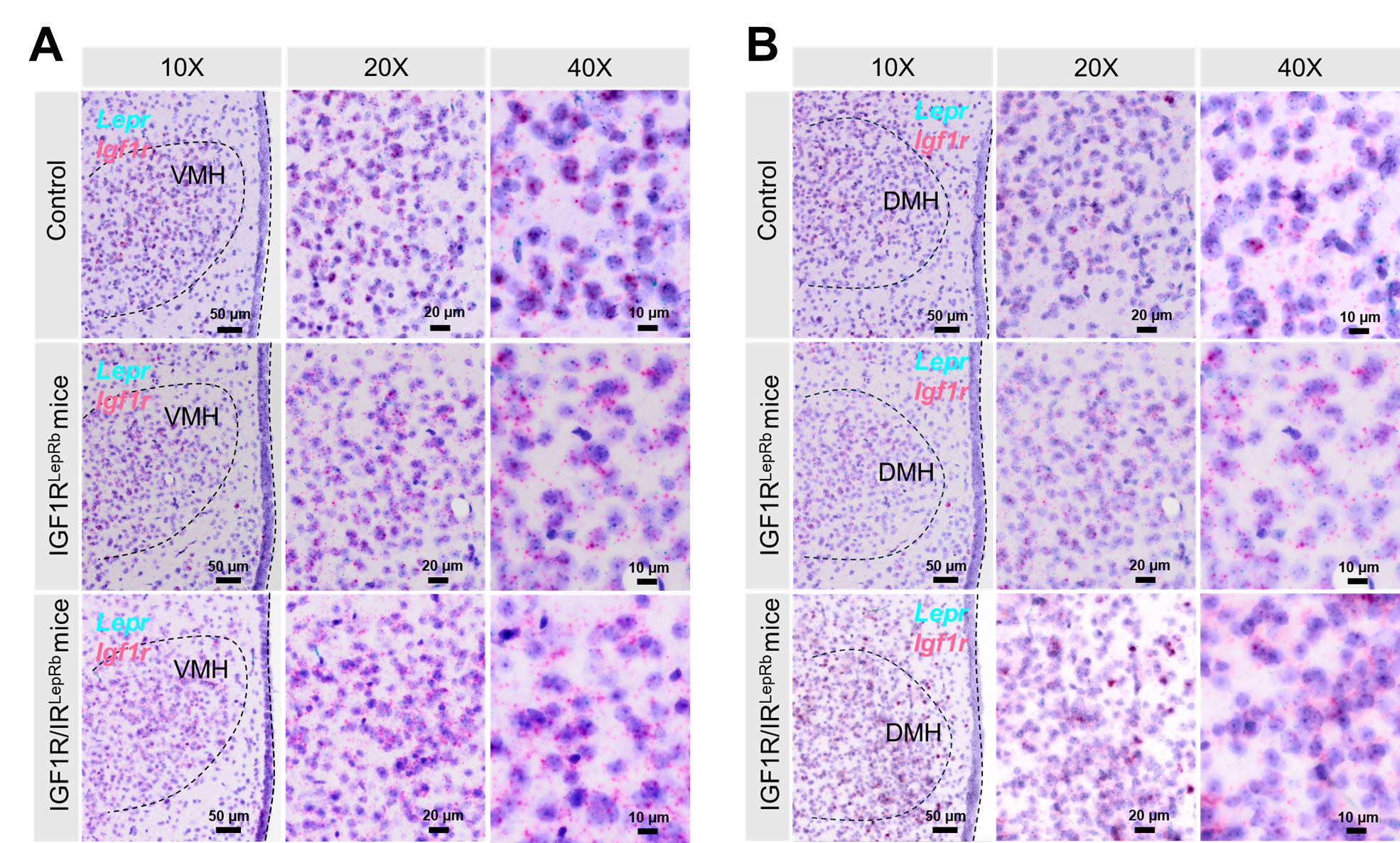
**

**Supplemental Figure 1. Representative BaseScope detection of *Lepr* and *Igf1r* mRNA colocalization in the VMH and DMH.**

**
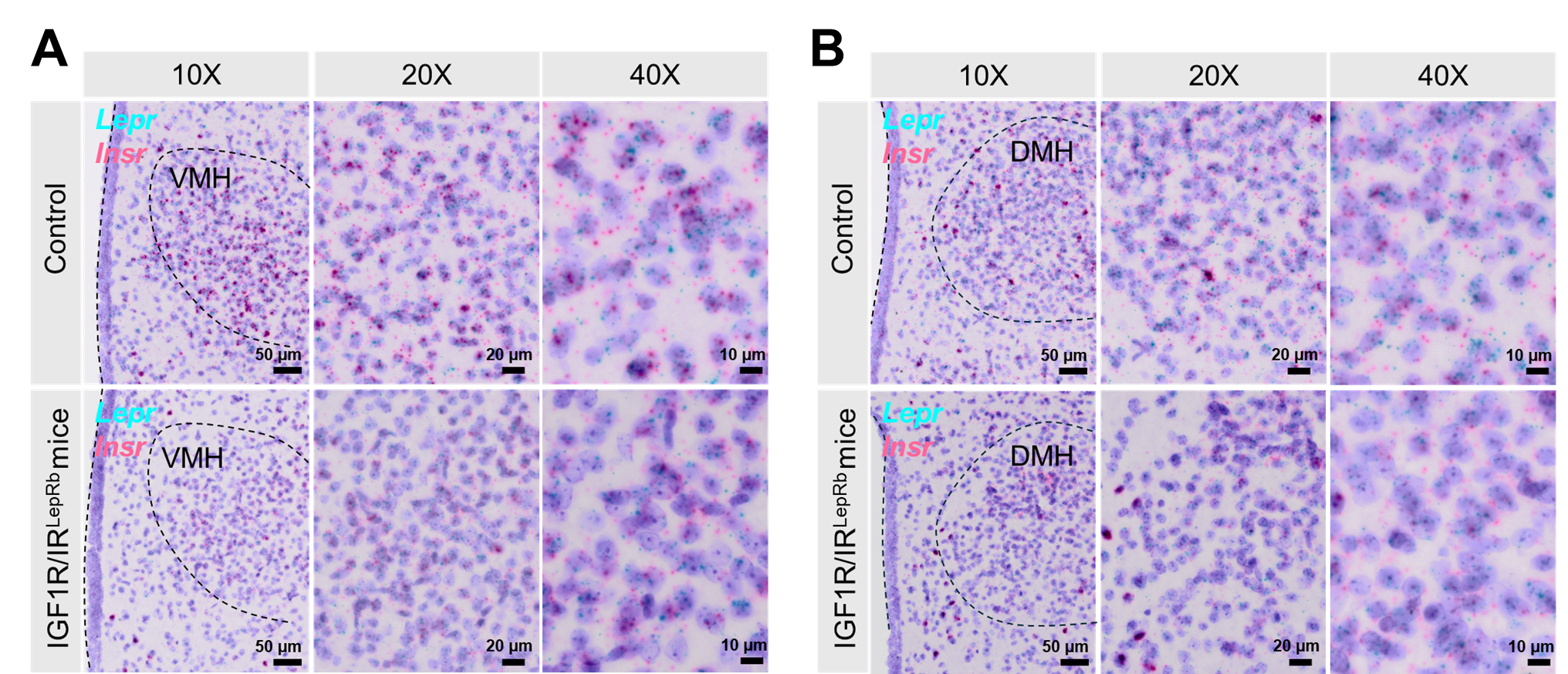
**

**Supplemental Figure 2. Representative BaseScope detection of *Lepr* and *Insr* mRNA colocalization in the VMH and DMH.**

**
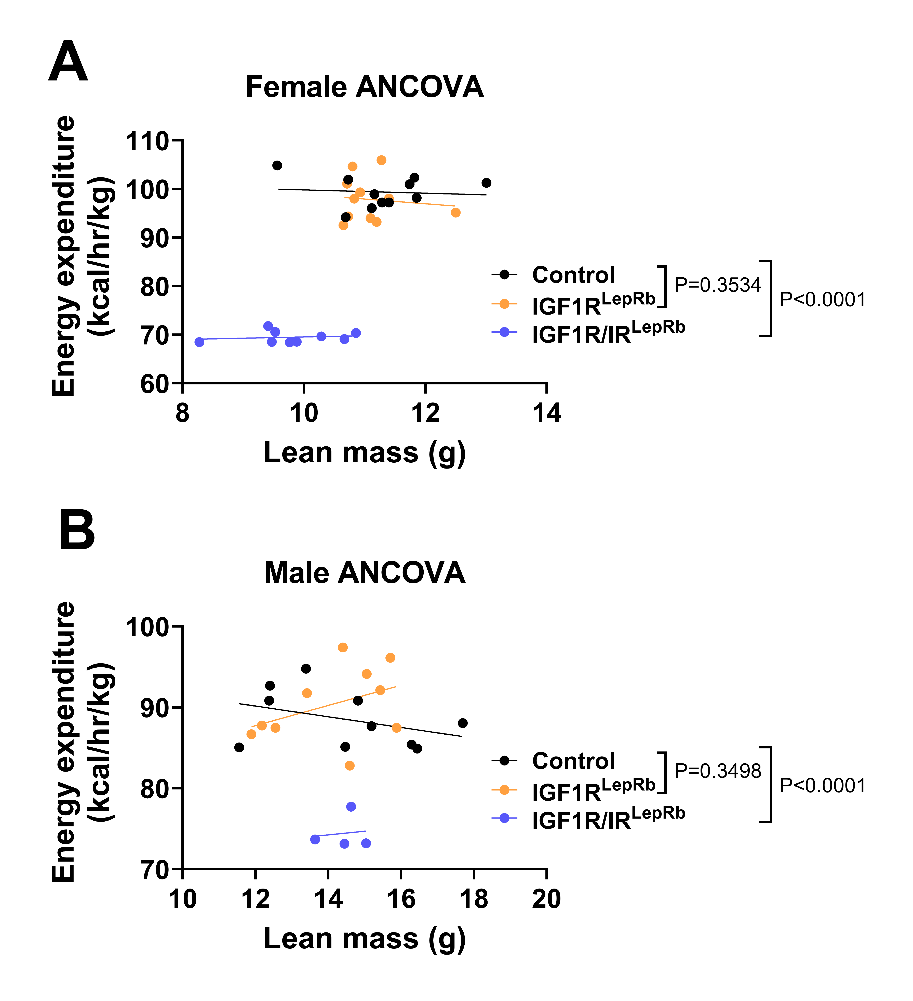
**

**Supplemental Figure 3. ANCOVA analysis of energy expenditure adjusted by lean mass.**

**
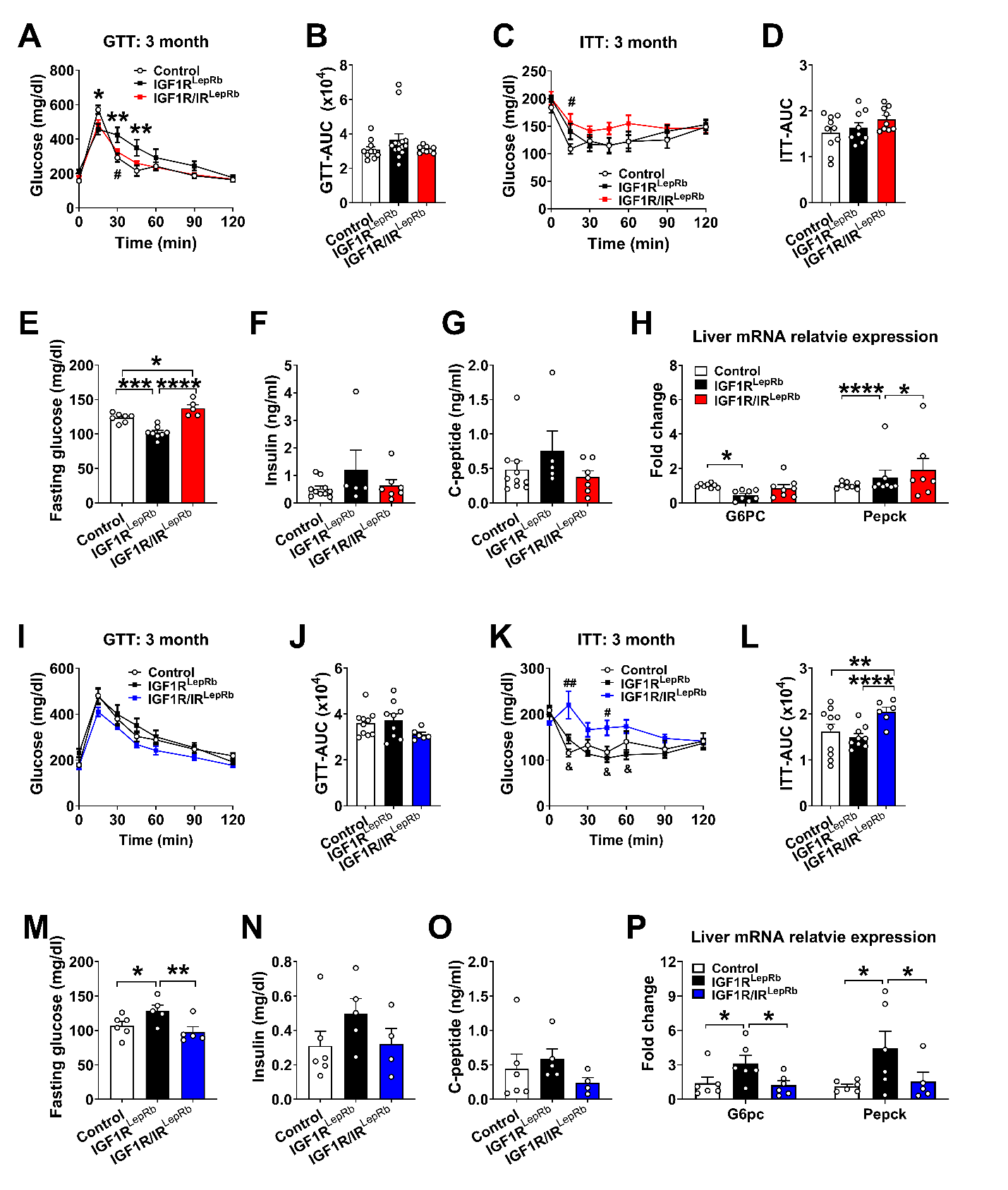
 Supplemental Figure 4. Changes in glucose homeostasis in IGF1R^LepRb^ and IGF1R/IR^LepRb^ mice**.

**Supplemental Table 1. Summary of primers.**

| **Primer** | **Sequence 5’-3’** |
| --- | --- |
| IGF1R forward | CTTCCCAGCTTGCTACTCTAGG |
| IGF1R reverse | CAGGCTTGCAATGAGACATGGG |
| IGF1R delta | TGAGACGTAGCGAGATTGCTGTA |
| IR forward | TGCACCCCATGTCTGGGACCC |
| IR reverse | GCCTCCTGAATAGCTGAGACC |
| IR delta | TCTATCATGTGATCAATGATTC |
| ADRB3 forward | TCGACATGTTCCTCCACCAA |
| ADRB3 reverse | GATGGTCCAAGATGGTGCTT |
| Cidea forward | AGGGAGGGACCTTAGGGAAT |
| Cidea reverse | CCAAGTCCAGCTTGGTGAAT |
| PRDM16 forward | CCTAACTTTCCCCACTCCCTCTA |
| PRDM16 reverse | GCTCAGCCTTGACCAGCAA |
| PPARγ forward | AGCCGTGACCACTGACAACGAG |
| PPARγ reverse | GCTGCATGGTTCTGAGTGCTAAG |
| G6PC forward | GGCTCACTTTCCCCATCAGG |
| G6PC reverse | ATCCAAGTGCGAAACCAAACAG |
| Pck1 forward | CCCACTGGGAACACAAACTT |
| Pck1 reverse | CCTTTCTTCTCTTTGGATGATCT |

**Supplemental Table 2. Summary of phenotypic changes in IGF1R^LepRb^ and IGF1R/IR^LepRb^ mice.**

| **Females** | | | | | |
| --- | --- | --- | --- | --- | --- |
|  | **Parameter** | **IGF1R^LepRb^** | **IGF1R/IR^LepRb^** | **IR^LepRb^** | **p110^LepRb^** |
| **Reproduction** | Vaginal opening | **↓** | **↓** | **→** | **↓** |
|  | First estrus | **↓** | **↓↓** | **↓** | **↓** |
|  | Fertility | **↓** | **↓** | **→** | **→** |
|  | Numbers of pups per litter | **↓** | **↓** | **→** | **→** |
|  | Ovarian follicles | **↓** | **↓** | NA | NA |
| **Growth** | Body length | **↓** | **↓↓** | **→** | **↓** |
|  | Bone mass | **↑** | NA | NA | NA |
| **Metabolism** | Body weight | **↓** | **↓** | **→** | **↓** |
|  | Fat / lean mass | **→** / **→** | **↑** / **↓** | NA | NA |
|  | Food intake | **↓** | **↓** (trend) | **→** | NA |
|  | Energy expenditure | **→** | **↓** | **→** | NA |
|  | Locomotor activity | **↑** | **↓** | **→** | NA |
|  | Thermogenesis genes | **↑** | **↓** | NA | **→** |
|  | Fasting glucose | **↓** | **↑** | NA | **→** |
|  | Gluconeogenic genes | **↓** | **→** | NA | NA |
| **Males** | | | | | |
| **Reproduction** | Balanopreputial separation | **↓↓** | **↓** | NA | **→** |
|  | First date of conception | **↓** | **↓** | NA | NA |
|  | Fertility | **↓** | **↓** | NA | **→** |
|  | Numbers of pups per litter | **↓** | **↓↓** | NA | **→** |
|  | Spermatogenesis | **↓** | **↓** | NA | NA |
| **Growth** | Body length | **↓** | **↓** | **→** | **↓** |
|  | Serum IGF-1 / GH levels | **↓** / **↓** | **→** / ↓ | NA | NA |
|  | Bone change | **↑** | NA | NA | NA |
| **Metabolism** | Body weight | **→** | **→** | **→** | **↓** |
|  | Fat / lean mass | **→** / **→** | **↑** / **↓** | NA | **↓** / **↓** |
|  | Food intake | **→** | **→** | **→** | **↑** |
|  | Energy expenditure | **→** | **↓** | **→** | **↑** |
|  | Locomotor activity | **↓** | **↓** | **→** | **↑** |
|  | Insulin tolerance | **→** | **↓** | **→** | **→** |
|  | Fasting glucose | **↑** | **→** | **↓** | **→** |
|  | Gluconeogenic genes | **↑** | **→** | NA | NA |
